# Probabilistic operational management of a renewable-based microgrid considering uncertainties using the self-adaptive gravitational search algorithm

**DOI:** 10.1038/s41598-026-42839-8

**Published:** 2026-03-05

**Authors:** Emad M. Ahmed, Zaki A. Zaki, Mehrdad Ahmadi Kamarposhti, El Manaa Barhoumi, Ilhami Colak

**Affiliations:** 1 Department of Electrical Engineering, College of Engineering, Jouf University, Sakaka, 72388 Saudi Arabia; 2 Department of Electrical Engineering, Jo.C., Islamic Azad University, Jouybar, Iran; 3 Department of Electrical and Computer Engineering, College of Engineering, Dhofar University, Salalah, Oman; 4 Department of Electrical and Electronics Engineering, Faculty of Engineering and Applied Sciences, Istinye University, Istanbul, Turkey

**Keywords:** Microgrid energy management, Self-adaptive gravitational search algorithm (SGSA), Uncertainty modeling, Point estimation method (PEM), Battery storage, Energy science and technology, Engineering

## Abstract

Increasing uncertainties in electricity prices, load demand, and renewable energy generation pose significant challenges for optimal microgrid operation in deregulated electricity markets. This paper proposes a self-adaptive Gravitational Search Algorithm (SGSA), which enhances the standard GSA by incorporating a self-adaptive mutation operator with two movement strategies to mitigate premature convergence and improve solution quality. To model uncertainties in load demand, market prices, and renewable outputs, the 2 m-Point Estimation Method (PEM) is employed as a computationally efficient alternative to conventional stochastic approaches. The proposed SGSA-PEM framework is applied to a low-voltage microgrid consisting of microturbines, phosphoric acid fuel cells, photovoltaic units, wind turbines, and battery storage. Simulation results indicate that the integration of battery storage reduces the total generation cost by up to 49.7%, while renewable energy penetration increases by approximately 10% during peak demand periods. Furthermore, comparative analysis shows that SGSA achieves lower operating costs and converges about 25% faster than standard GSA and Particle Swarm Optimization (PSO). The results confirm that the proposed framework provides a computationally efficient and robust solution for probabilistic microgrid energy management under uncertainty.

## Introduction

Microgrids are small-scale energy systems that use distributed energy resources (DER), which are made of microturbines, phosphoric acid fuel cells, photovoltaic (PV) units, wind turbines, battery storage, and others to power local loads, while multiple agents including generators, consumers, or system operators could coordinate by using of centralized or distributed agent (e.g. Microgrid Central Controller or MGCC)^1–3^. Microgrids have the ability to operate in the normal mode (grid-connected) or in an islanded operation mode so that the grid can supply maximum flexibility for energy management. In a competitive electricity system, microgrid operations can be faced with uncertainty regarding market prices, load demands, and renewable expectations due to the stochastic characteristics of wind speeds and solar irradiance processes. Other uncertainties which could complicate reliable and cost-effective operations are fuel prices, random price signals, etc. All of these uncertainties would have a requirement for advanced optimization approaches to model and orchestrate distributed generators (DG) and provide stabilization with their networks^1,4^.

Recent research focuses on the increasing prospects of microgrid technologies to accommodate a high degree of intermittent renewable energy while ensuring stability and flexibility. The importance of stability improvement using methodologies like “control-oriented strategies” is acknowledged for improving the stability of PV-wind-based hybrid systems under various conditions^5^. Efficient scheduling and energy management techniques have been presented to deal with various uncertainties and address multi-objective microgrid scenarios while ensuring the reliability of operation^6^. In addition, methods for identifying causes and sources of voltage sags in systems with high renewable penetration can help in planning and improving reliability^7^. In addition to these methodologies, robust optimization techniques can efficiently handle complex microgrid scenarios involving various uncertainties and non-linearities while ensuring the reliability of operation^8^.

Microgrid energy management is the process of optimizing the dispatch of distributed energy resources (DER) to minimize cost while respecting load and grid limitations. The best example of traditional type methods is the optimal power flow (OPF) method that tells the operator how to optimize the dispatch of active and reactive power by defining those active power supply limits related to steady state and using price signals to generate bids that increase generation outputs or decrease the microgrid’s non-critical loads related to the real-time market^2^. The microgrid system operator (SO) or grid operator would look at the mock real-time price bids in the market to decide the output levels of generators when the economic operation of DERs can take place^9^.

For instance, in an islanded condition, shown in^2^, a microgrid will disconnect from the main grid and rely on a flywheel energy storage system (FESS) to supply the loads in the islanded microgrid. The microgrid controller (MGCC) will be responsible to control the dispatch of distributed generation (DG) of power, providing flywheel support without exceeding operating limits, until flywheel output is no longer feasible. The classical OPF will consider adequate operation of DGs either by estimated economic costs or reliability estimates, but will always be strictly based on the assumption that every input data point to the model is true for any condition, which is unlikely in an open access market with constantly shifting prices or the even more challenging switching costs of variable fuel and renewable power operating conditions across the region^2,9^.

Neither deterministic methods for optimal power flow (OPF), nor heuristic algorithms such as Gravitational Search Algorithm (GSA), Particle Swarm Optimization (PSO), and genetic algorithms consider any uncertainties in market prices, load demands, or renewables generation, resulting in emergencies or suboptimal dispatch decisions. Probabilistic methods e.g. Monte Carlo simulations (MCS), are the most accurate but very expensive from a computational point of view if there are many iterations required. Simplified methods are computationally inexpensive, but they also sacrifice accuracy. Traditional heuristics may exhibit premature convergence or become ‘stuck’ in local optima, especially with varying market conditions. Additionally, Distributed Generation (DG) integration has led to additional complexities, which can cause challenges in existing protection systems, harmonics, reduced short circuit impedance, and coordination. These present major challenges for the operation and safety of the network, especially while in islanded operation^10^.

Providing accurate price indications at all times and for all applications remains challenged. The upgrade of the system for new loads could be initiated, delayed, or not allowed altogether due to microgrid integration and to radial distribution systems. The objective of price indications is to provide justified investment in generation or load control when applicable loads are congested. In principle this is a good proposal and will instill efficiency, in practice this is difficult to achieve. Active resource management is required for the microgrid, to active control several pieces of information and their management time scales are considered. For example, generators be controlled in milliseconds to seconds for safety and stability. To determine what is the actual electricity in and out, it may take a few minutes. Although energy storage (possible concerns) may take a few days to consider. In this sense, the microgrid aims to eliminate unnecessary excessive charges. The system operator (SO) sets generator levels, based on offered prices in the electricity market. It is the generator’s responsibility to determine the services based on the market price. The generation continues, but it is the SO who will see the price conditions change, when the order of the day is presented. The price change will influence a generator’s outputs and loads to reduce inappropriate loads, if necessary, to provide balance. This method does in some consider as the price indication based on physical response^2,9^.

A steady-state AC power optimization based optimal power flow (OPF) algorithm was proposed in^11^ to implement the microgrid economic operation in real time in the market. To ensure economic operation, OPF minimizes costs for active and reactive power plus load shedding, while being subject to generation limits, load constraints, voltage limits, and other automatic control systems. One scenario is where the microgrid operates in island mode. At *t = 0* seconds, a fault occurs on the connected grid and isolates the microgrid with a circuit breaker. The flywheel energy storage system serves loads during the islanding transition. The flywheel then submits a price signal to the Microgrid Central Controller (MGCC). In turn, the MGCC implements the OPF and communicates the instructions and signals to the DGs. The micro-generators react to the MGCC signal with electricity output increased (and price increase) before the flywheel fully discharges and while the DGs ramp-up power. Ultimately, this helps demonstrate new signals from the MGCC change, allowing the DGs to run before depletion of the flywheel. Unknown parameters affect electricity markets, including electricity price, fuel price, wind speed, or sunlight. These increase electricity price or supply. With random price signals, microgrid partners cannot participate actively^1,11^.

Microgrid-related decisions with respect to distributed generation (DG) operation and consistent operational spend should estimate electricity and fuel price parameters^1,4^. Gas price and electricity price profiles have been calculated based on the estimated parameters in order for microgrid partners to establish an investment or operational financial threshold^1^. For example, a higher gas price means reduced investment. Despite their inherent advantages, DG has inherent negative effects on distribution networks, including voltage levels impacted due to DG and overall coordination of protection. This process makes it more cumbersome to develop and operate network protection^12^. Connection of the DG can lead to harmonics, and lower short circuit impedance for protection of circuits. Islanded DG during the shut down period can create unsafe conditions for repairmen^1^.

The asymmetric two-point estimation methods, such as US2PEM, recognize that various uncertainties from renewable sources referred to as generation, including both wind turbines and solar systems, as well as from load demand, manifest as input variables. This is why it is advisable to apply two-point estimation approaches when there is a situation with input as well as output variable uncertainties, where the probabilistic distribution is recognized with regard to an effective energy management solution for microgrids^13,14^.

The output variables considered for US2PEM included near-term (active) power loss changes and (reactive) power input from the main grid. Probability methods can be classified into three groups or categories: Monte Carlo simulation (MCS) methods, analytical methods, and approximate methods. Monte Carlo simulation methods are simple to implement and provide accurate outputs, however, often at a high computation cost. Analytical methods require fewer computations as they make use of complex calculations to estimate uncertainty (though not always the outputs). Approximate methods allow for a balance between computational efficiency and quality accuracy^15^.

The 2 m-Point Estimation Method (PEM) is an approximate method that serves the purpose of modeling uncertainty in electrical systems in an effective way^15^. Although 2 m PEM obtains a statistical moment of output random variables using a deterministic methodology, it requires fewer simulations than the Monte Carlo methodology. Gravitational Search Algorithm (GSA) is based on gravity and mass interacting with one another^16^. GSA is related to a Central Force Optimization (CFO) algorithm^17^. It differs in terms of the mechanism used, as CFO uses inverse linear distance over an inverse squared distance of gravity. In GSA, the masses interact according to laws of Newtonian gravity and motion.

Investigations into microgrid energy management have received considerable research attention, but previous literature suggests the methods explored in previous literature perform poorly under uncertainty. Gao et al.^5^ proposed a genetic algorithm-based energy management controller for deterministic microgrid operations, without accounting for stochastic renewables or market fluctuations. Similarly, Sortomme and El-Sharkawi^18^ presented a microgrid optimization procedure that implemented Optimal Power Flow (OPF) and Particle Swarm Optimization (PSO) that was able to obtain reductions of cost and peak load shaving up to 14% and 10 MW over a 48 h timeframe respectively. Again, the use of fixed parameters associated with PSO is problematic and causes restrictions for the utility of the PSO function - premature convergence is possible when dealing with the attendant uncertainty introduced to the optimization problem which ultimately leads to less robust results.

Investigations into microgrid energy management have received considerable research attention, but previous literature suggests the methods explored in prior studies perform poorly under uncertainty^1,5,18^. Gao et al.^6^ proposed a genetic algorithm-based energy management controller for deterministic microgrid operations, without accounting for stochastic renewables or market fluctuations. Similarly, Sortomme and El-Sharkawi implemented an optimization procedure that used Optimal Power Flow (OPF) and Particle Swarm Optimization (PSO), which was able to achieve reductions of cost and peak load shaving up to 14% and 10 MW over a 48-hour timeframe, respectively^5,18^. Again, the use of fixed parameters associated with PSO is problematic and causes restrictions for the utility of the PSO function — premature convergence is possible when dealing with the attendant uncertainty introduced to the optimization problem, which ultimately leads to less robust results^10,16^.

Reference^9^ investigated a fuel-conservation problem for electric and thermal energy services, not considering the stochastic behavior of renewables, which could exacerbate inefficiencies. Tsikalakis and Hatziargyriou^19^ developed an optimization for the operation of grid-connected microgrids with a central controller that participates in real-time markets, but their deterministic-based operational model relies on stable and predictable conditions that may be impossible to assume when markets become volatile. Chedid et al.^20^ investigated optimal design of a university campus microgrid under unreliable grid conditions, considering PV generation and battery storage within an economic optimization framework.

Recent progress has been made in optimizing the use of microgrids. Fadlullah et al.^5^ provided energy management systems in microgrids and optimization and control strategies while providing analysis on the computational errors of stochastic renewables and load demands. Kharrich et al.^21^ provided an optimization algorithm for hybrid systems with photovoltaic/wind/diesel/biomass/battery that makes use of a travelling damped wave algorithm, and showed that overall efficiency was constrained by the limitations made regarding the distribution of uncertainty. Dayalan and Rathinam^22^ proposed a more reliable energy management with demand response that requires further improvements in computing efficiency for real-time application. Similarly, Dai et al.^23^ proposed an optimized energy management approach for multi-energy multi-microgrid networks that leverages a mountain gazelle optimizer, and allows for additional cost and emissions savings through decentralized control and more frequent real-time updates. Akter et al.^24^ conducted a comprehensive review of meta-heuristic algorithms for optimizing microgrid operations, and noted that genetic algorithms and particle swarm optimization algorithms are stuck in frozen local optima under transient conditions and refinements are needed.

The studied research illustrates the difficulty in effectively dealing with uncertainty associated with environmental changes. Deterministic approaches^25^ under-perform in changing markets, and statistical techniques such as the Monte Carlo model from reference^26^ are too computationally intensive to use. We propose SGSA and PEA approaches, which do not suffer from the problems described above. PEM effectively models uncertainties in the electrical loads, market prices, and renewable output using statistical moments^15^. SGSA’s self-adaptive mutation operator uses two dynamically selected movement strategies produced via a probabilistic model that outperforms fixed-parameter heuristics (e.g., particle swarm optimization and differential evolution) and was shown to reduce costs in simulation experiments^10^. This tool enables reliable, efficient, low-cost microgrid operations under uncertainty.

However, even though precision optimization methods, as mentioned above, i.e., Mixed Integer Linear Programming methods, can ensure the optimality of the solutions obtained for not only the linear optimization problems but also for the convex optimization problems, as mentioned above, the nonlinearity of the energy management problem, as discussed above, and the randomness due to the incorporation of the renewable and storage components of the energy management problem through the application of the stochastic models, as mentioned above, if considered while obtaining the optimization results, would also imply significant simplifications, as discussed above. Thus, an ideal approach of making use of the metaheuristic optimization algorithm to obtain a high degree of near-optimal solutions.

In summary, the study addresses some of the important research gaps in microgrid optimization problems under uncertainty, where traditional methods might be computationally burdensome and normally used stochastic approaches like Monte Carlo require high resources. In order to fix both difficulties, we present a novel approach in this paper, namely combining selfadaptive Gravitational Search Algorithm and 2 m-Point Estimation Method (PEM). The main novelties and contributions of this work are:

- Development of self-adapting GSA: Propose a mutation operator for efficient quality solutions and convergence rates for microgrid optimization problems.
- Efficient uncertainty modeling: Incorporating PEM helps in representing uncertainties of load demand, market prices, and RE generation with significantly less simulations run using traditional Monte Carlo simulations.
- Comprehensive microgrid application: The methodology will be applied to a low voltage microgrid which incorporates units of microturbines, phosphoric acid fuel cells, photovoltaic units, wind units, as well as battery storage.
- Operational Performance Improvement: The comparision results in the paper show that the SGSA-PEM has achieved lower costs, increased renewable penetration, and faster convergence than the standard metaheuristics such as GSA and PSO.

The present study thus proposes a computationally efficient, viable approach to probabilistic microgrid planning and operation, with a highlight on the potential of using SGSA as a strong metaheuristic optimization technique for power and energy systems.

The structure of this paper consists of six sections: the objective function and constraints are discussed in “Objective function and microgrid constraints”, the 2 m-Point Estimation Method is introduced in “2m-point estimation method”, “Self-adaptive gravitational search algorithm” discusses the self-adaptive Gravitational Search Algorithm, in “Simulation and analysis of results” we apply our proposed method to an example network showing two scenarios to help make our case, and then we conclude in “Conclusion” giving results and suggestions.

## Objective function and microgrid constraints

The total energy amount and the operating amount of the micro-grid include the fuel amounts of the units as well as the amounts of turning on/off these units.

The objective function can be written as follows^26^:

1 $$\begin{aligned} Min\mathop f\limits^{\sim } (X)&=\sum\limits_{{t=1}}^{{NT}} {\operatorname{Cos} {t^t}} \\ &= \sum\limits_{{t=1}}^{{NT}} {\{ \sum\limits_{{i=1}}^{{{N_g}}} {[u_{i}^{t}p_{{Gi}}^{t}B_{{Gi}}^{t}+Star{t_{Gi}} \times \hbox{max} (0,u_{i}^{t} - u_{i}^{{t - 1}})} } +Shu{t_{Gi}} \times \hbox{max} (0,u_{i}^{{t - 1}} - u_{i}^{t})] \\ & \quad +\sum\limits_{{j=1}}^{{{N_s}}} {[u_{j}^{t}p_{{sj}}^{t}B_{{sj}}^{t}} +Star{t_{sj}} \times \hbox{max} (0,u_{j}^{t} - u_{j}^{{t - 1}})+Shu{t_{sj}} \times \hbox{max} (0,u_{j}^{{t - 1}} - u_{j}^{t})+p_{{Grid}}^{t}B_{{Grid}}^{t}\}\end{aligned}$$

where:

*f*: estimated amount; NT: total number of hours.

Ng – Ns: The total number of production and storage units.

${p}_{Gi}^{t},{p}_{Sj}^{t}:$Active power output of the i-th generator and the j-th storage device.

${p}_{Grid}^{t}:$ Active power purchased from/sold to the energy supply unit at time *t*.

${u}_{i}^{t}$ : Unit status per hour.

${B}_{Gi}^{t},{B}_{Sj}^{t}:$The i-th DG source and the j-th storage device at hour t.

${B}_{Grid}^{t}$ : Suggested energy supply unit per hour.

X=[X_1_ × _2_ …X_t_ …X_NT_] Xt are state variable vectors that include the active powers of the units and their respective states, which can be defined as follows:

2 $${X^t}=[p_{{G1}}^{t},p_{{G2}}^{t},...,p_{{GNg}}^{t},...,p_{{sNs}}^{t},u_{1}^{t},u_{2}^{t},...,u_{{Ns+Ng}}^{t}]$$

The limitations of the problem are as follows:

*Power balance*:

3 $$\sum\limits_{{i=1}}^{{{N_g}}} {p_{{Gi}}^{t}+\sum\limits_{{j=1}}^{{{N_s}}} {p_{{sj}}^{t}+p_{{Grid}}^{t}=\sum\limits_{{D=1}}^{{{N_D}}} {p_{{{L_D}}}^{t}} } }$$

where PL_D_ is equal to the load level D.

*Real power generation capacity*:

4 $$\begin{gathered} p_{{Gi,\hbox{min} }}^{t} \leqslant p_{{Gi}}^{t} \leqslant p_{{Gi,\hbox{max} }}^{t} \hfill \\ p_{{sj,\hbox{min} }}^{t} \leqslant p_{{sj}}^{t} \leqslant p_{{sj,\hbox{max} }}^{t} \hfill \\ p_{{Grid,\hbox{min} }}^{t} \leqslant p_{{Grid}}^{t} \leqslant p_{{Grid,\hbox{max} }}^{t} \hfill \\ \end{gathered}$$

where: $P_{{G,\hbox{min} }}^{t},P_{{G,\hbox{max} }}^{t}$ : Minimum and maximum production of active power of i-th DG at hour *t*.

$P_{{S,\hbox{min} }}^{t},P_{{S,\hbox{max} }}^{t}$ : Minimum and maximum production of active power j of storage at hour *t*.

$P_{{grid,\hbox{min} }}^{t},P_{{grid,\hbox{max} }}^{t}$ : Minimum and maximum production of active power of the energy supply unit at hour *t*.

*Revolving reservation*:

5 $$\sum\limits_{{i=1}}^{{{N_g}}} {u_{i}^{t}p_{{Gi,\hbox{max} }}^{t}+\sum\limits_{{j=1}}^{{{N_s}}} {u_{j}^{t}p_{{sj,\hbox{max} }}^{t}+p_{{Grid.\hbox{max} }}^{t} \geqslant \sum\limits_{{D=1}}^{{{N_D}}} {p_{{{L_D}}}^{t}+\operatorname{Re} {s^t}} } }$$

where Res^t^ is equal to scheduled revolving reservation at time *t*.

*Limitations of energy storage*:

Since there are some limitations regarding the charging and discharging speed of storage devices during each time period, the following equation and limitations can be considered:

6 $$W_{{ess}}^{t}=W_{{ess}}^{{t - 1}}+{\eta _{ch\arg e}}{P_{ch\arg e}}\Delta t - \frac{1}{{{\eta _{disch\arg e}}}}{P_{disch\arg e}}\Delta t$$

7 $$\begin{gathered} W_{{ess,\hbox{min} }}^{t} \leqslant W_{{ess}}^{t} \leqslant W_{{ess,\hbox{max} }}^{t} \hfill \\ {P_{ch\arg e,t}} \leqslant {P_{ch\arg e,\hbox{max} }};{P_{disch\arg e,\hbox{max} }} \leqslant {P_{disch\arg e,\hbox{max} }} \hfill \\ \end{gathered}$$

where:

$W_{{ess}}^{t},W_{{ess}}^{{t - 1}}$ : Battery energy storage at time *t* and *t-1*.

P_Charge, max_ : the maximum charging rate during the specified period of time.

P_discharge, max_: the maximum discharge rate during the specified time period.

${\eta _{ch\arg e}}({\eta _{disch\arg e}})$ : Battery charge (discharge) efficiency.

W_ess, min_ (W_ess, max_): upper and lower limits of battery energy storage.

## 2m-point estimation method

In 1975, Rosenblueth proposed the 2 m PEM method for solving probability problems. The Rosenblueth method was inefficient due to the large number of simulations required^27^. In 1989, Harr proposed a new PEM to overcome the shortcomings of the Rosenblueth method^15^. Although Harr’s method was computationally more efficient than previous methods, it is limited to symmetric variables^15^. Hong introduced an effective point estimation method suitable for both symmetric and asymmetric variables^28^. This paper uses 2 m PEM proposed by Hong to model the uncertainty in load demand, market prices and available output power of wind turbine and photovoltaic units.

In this study, the uncertainties concerning the availability of renewables and the load demands are managed using the 2 m Point Estimation Method (PEM). This technique strikes a reasonable balance in terms of efficiency in computation. Compared to the earlier-recommended approaches such as stochastic programming and robust optimization along with other probabilistic methods to manage uncertainties, the PEM boasts faster computation with reliable modeling. The recent developments in multi-timescale stochastic optimization for managing uncertainties, along with data-model-based acceleration for large-scale network-constrained unit commitment problems, and set-based interval optimization for integrated energy systems operation efficiently in uncertain conditions are worth mentioning^29–31^.

Mathematically, the management of utilization and definite energy of micro-grids can be shown as follows:

8 $$S=f(\nu ,c)$$

where v is the set of input variables, S is the output of the exploitation and energy management problem, and f is the set of energy and operation amount equations. In order to solve the deterministic EOM problem, all input random variables (IRVs) were considered equal to their predicted values. However, the actual values ​​for some variables may be different from their predicted values​​^10^, including the errors in the predicted output powers for wind turbine and photovoltaic units. The f function transfers the uncertainty from the IRVs to the output variable. Considering m to IRV, Eq. (8) can be written as follows:

9 $$S=f(c,{z_1},{z_2},...,{z_m})$$

where c is the set of specific variables, and z_l_ (l = 1,.,m) are the input variables under uncertainty.

The idea behind PEM is to calculate the statistical information of the output variables using a set of deterministic energy and utilization management problem solutions for a small number of estimated values ​​of IRVs. In order to find the statistical moments of the random output variable, 2 m PEM requires only a small number of the first principal moments of the IRVs (coefficients of mean, variance, deviation. This feature is a significant advantage of the point estimation method in which achieving the implementation of IRVs features is a difficult task^32^.

The 2 m Point Estimation Method (PEM) generates two possible concentrations for each input random variable (IRV), z_l_​, as as $({\mathrm{z}}_{\mathrm{l},1},{\mathrm{w}}_{\mathrm{l},1}$) and (${z}_{l,2},{\omega}_{l,2})$, where po = 1,2. Here, z_l, po_​ is the po-th location of z_l_​ and ω_l, po_​ is the weight coefficient that determines the importance of the corresponding location in evaluating the statistical moments of the random output variable. The EOM is simulated 2 m times in the proposed probabilistic method. In each simulation, one of the IRVs is fixed in one of its locations and the other IRVs are equal to their average value, so we have:

10 $${S_{(l,po)}}=f(c,{\mu _{{z_1}}},{\mu _{{z_2}}},...,{z_{l,po}},...,{\mu _{{z_m}}})$$

where and the specified IRV locations, and the average value of the remaining IRVs. When the 2 m deterministic solutions of EOM, S(l, po) are determined, the mean and standard deviation of the random output variable can be estimated^10,32^.

The 2 m PEM step-by-step process for calculating the moments of the output random variable is summarized as follows:

*Step 1*: Determine m (the number of input random variables of the estimation method is 2 m points).

*Step 2*: Choose an unknown parameter z_l_ (Zl is equal to the value of the lth random input variable of the 2 m point estimation method).

*Step 3*: Set E(Sh) = 0, h = 1,2.

*Step 4*: The deviation () related to is calculated based on the following Eq. 

11 $${\lambda _{{z_1},3}}=\frac{{E\left[ {{{({z_1} - {\mu _{{z_1}}})}^3}} \right]}}{{{\sigma _{{z_1}}}}}$$

where $E[{({z_l} - {\mu _{zl}})^3}]\sum\nolimits_{{j=1}}^{N} {{{({z_{l,j}} - {\mu _{zl}})}^3}} \times \Pr ob({z_{l,j}})$N is the number of observations and the probability of each observation determined by the operator system^10^.

*Step 5*: Two standard locations are calculated as follows.

12 $${\xi _{(l,po)}}=\frac{{{\lambda _{{z_1},3}}}}{2}+{( - 1)^{3 - po}}\sqrt {{{(m+{{\left( {\frac{{{\lambda _{{z_1},3}}}}{2}} \right)}^2})}^{}}} \begin{array}{*{20}{c}} ,&{po=1,2} \end{array}$$

*Step 6*: The two estimated locations are calculated as follows.

13 $${Z_{l,k}}={\mu _{{z_1}}}+{\xi _{{z_1},k}}.{\sigma _{{z_1}}}\begin{array}{*{20}{c}} {}&{po} \end{array}=1,2$$

*Step 7*: The definitive EOM for the estimated location is calculated.

14 $${S_{(l,po)}}=f({\mu _{{z_1}}},{\mu _{{z_2}}},...,{z_{l,po}},...,{\mu _{{z_m}}})\begin{array}{*{20}{c}} {} \\ {} \end{array}\begin{array}{*{20}{c}} {po=1,2} \\ {l=1,2,..,m} \end{array}$$

*Step 8*: The two weighting coefficients z_l are calculated as follows (*po* equals the second weighting coefficient Zl, po).

15 $${\omega _{l,po}}=\frac{{{{( - 1)}^{po}}}}{m}\frac{{{\xi _{l,3 - po}}}}{{{\xi _{l,1}} - {\xi _{l,2}}}}\begin{array}{*{20}{c}} {}&{po=1,2} \end{array}$$

*Step 9*: The first and second time of the output random variable (total energy and operating amount) are updated.

16 $$E({S^h})=E({S^h})+\sum\limits_{{po=1}}^{2} {{\omega _{l,po}}.{{(s(l,po))}^h}} \begin{array}{*{20}{c}} {}&{h=1,2} \end{array}$$

*Step 10*: Steps 3–9 are repeated until all uncertain parameters are considered.

*Step 11*: The average and standard deviation of the total energy and the usage amount are calculated (µ_s, σ_s are the average and standard deviation of S).

17 $${\mu _s}=E({S^1}),\begin{array}{*{20}{c}} {}&{{\sigma _s}=} \end{array}\sqrt {E({S^2}) - {{(E({S^1}))}^2}}$$

The probability density function of the output random variable can be approximated and plotted using the calculated mean and standard deviation and the Gram–Charlier series method^10^.

Although various advanced frameworks for dealing with uncertainties, such as stochastic programming, robust optimization techniques, and Information Gap Decision Theory (IGDT), have been successfully used in various power system issues, in most cases, these methods have shown high computational complexities because of the necessity for large scenario sets, multi-stage formulation, and/or optimization nesting, etc., which may not be suitable for real-time decision-making issues in microgrid energy management.

In contrast, the 2 m-point estimation method is a computationally efficient approach that can be used due to its ability to approximately account for uncertainties through a small number of deterministic calculations, yet maintaining key statistical properties of initial random variables. Such a point is particularly useful when it comes to dealing with unique microgrid applications.

## Self-adaptive gravitational search algorithm

The Gravitational Search Algorithm (GSA), being simple in terms of structure, has found extensive utilization in solving continuous or nonlinear optimization problems due to balanced exploration and exploitation. Nevertheless, the original GSA was reported to have some drawbacks in solving challenging optimization problems due to early convergence and loss of population diversification. This fact calls for an enhanced self-adaptive version of GSA, as proposed in this study.

### Review of the standard gravity search algorithm

The Gravitational Search Algorithm (GSA), proposed by Rashedi et al.^16^, belongs to a family of population-based (meta-) heuristic optimization algorithms that are inspired by the laws of gravity and motion introduced by Newton. In GSA, each candidate solution (also called a particle) corresponds to a position in the search space and/or potential solution of the optimization problem. A mass is assigned to each particle that is proportional to the particle fitness, with heavier masses representing better solutions. The particles interact with each other through gravitational forces that give them momentum to move toward better or optimal solutions^16^.

In the proposed SGSA framework, each particle represents a complete candidate solution for the microgrid energy management problem. Mathematically, the j-th particle is defined as:

18 $${\mathrm{X}}_{{\mathrm{j}}} = \left[ {{\mathrm{P}}_{{{\mathrm{G1}}}} \left( {\mathrm{1}} \right), \ldots ,{\mathrm{P}}_{{{\mathrm{G1}}}} \left( {\mathrm{t}} \right), \ldots ,{\mathrm{P}}_{{{\mathrm{Gn}}}} \left( {\mathrm{t}} \right),|{\mathrm{P}}_{{{\mathrm{S1}}}} \left( {\mathrm{1}} \right), \ldots ,{\mathrm{P}}_{{{\mathrm{Sm}}}} \left( {\mathrm{t}} \right),|{\mathrm{P}}_{{{\mathrm{Grid}}}} \left( {\mathrm{1}} \right), \ldots ,{\mathrm{P}}_{{{\mathrm{Grid}}}} \left( {\mathrm{t}} \right)} \right]$$

where:

X_j_​: decision vector of particle j.

P_Gi_​​(t): output power of distributed generation unit i at time t, i = 1,2,…,n.

P_Sk_​​(t): charging/discharging power of storage unit k at time t, k = 1,2,…,m.

P_Grid_​(t): power exchanged with the main grid at time t.

t = 1,2,…,T: time step index in the planning horizon.

T: total number of time steps (e.g., 24 h).

This representation ensures that all variables influencing the microgrid operation are included in the particle, and SGSA can efficiently search for the optimal solution by updating particle positions according to gravitational forces derived from fitness values^16^.

The gravitational force between two particles *j* and *e* is stated as:

19 $$F_{{je}}^{{k,t}}={G^k} \times \frac{{M_{j}^{k} \times M_{e}^{k}}}{{R_{{je}}^{k}+\varepsilon }} \times (X_{j}^{{k,t}} - X_{e}^{{k,t}})$$

where:

G^k^ is Gravitational constant at iteration *k*, M_j_​ and M_e_​ are the masses of particles *j* and *e*,* R*_*je*_ is the Euclidean distance between them, *X*_*j*_​ and *X*_*e*_ are their positions. The gravity constant Gk decreases during the optimization process as follows:

20 $${G^k}={G^0} \times \exp \left( {\varpi \times \frac{{Iter}}{{Ite{r_{\hbox{max} }}}}} \right)$$

where:

Iter_max_: Maximum number of iterations.

Iter: existing iteration.

G_0_ and $\varpi$ are two constants that are set equal to 100 and 20, respectively^16^.

The mass value of each particle is calculated according to its fitting value using the following equations:

21 $$M_{j}^{k}=\frac{{Fit(X_{j}^{k}) - wors{t^k}}}{{bes{t^k} - wors{t^k}}}$$

22 $$M_{j}^{k}=\frac{{m_{j}^{k}}}{{\sum\nolimits_{{e=1}}^{{{N_{swrm}}}} {m_{e}^{k}} }}$$

where:

Fit (${X}_{j}^{k}$): Fit value of the j^th^ particle.

Nswrm: total number of bees in the swarm.

worst^k^ and best^k^: maximum and minimum value of fitting in repetition K (in minimization problem).

$M_{j}^{k},M_{e}^{k}$: Gravitational mass related to particles j and e.

In order to give the algorithm a random feature, the general gravitational forces, which are applied to the jth particles, are calculated as follows. The acceleration of each particle is defined as follows:

23 $$F_{j}^{{k,t}}=\sum\limits_{{e=1,e \ne j}}^{{{N_{swrm}}}} {{r_e} \times F_{{je}}^{{k,t}}}$$

Finally, the new location and velocity of each particle is updated by the following equations:

24 $$a_{j}^{{k,t}}=\frac{{F_{j}^{{k,t}}}}{{M_{j}^{{}}}}$$

25 $$V_{{new,j}}^{{k,t}}=a_{j}^{{k,t}}+r \times V_{{old,j}}^{{k,t}}$$

26 $$X_{{new,j}}^{{k,t}}=X_{{old,j}}^{{k,t}}+V_{{new,j}}^{{k,t}}$$

where:

$V_{{new,j}}^{{k,\tau }},V_{{old,j}}^{{k,\tau }}$ : The new and old speed of j th particle.

$X_{{new,j}}^{{k,\tau }},X_{{old,j}}^{{k,\tau }}$ : New and old solution j th particle.

In order to balance the search capability and represent the gravitational search algorithm, only a set of particles with the best proportional values ​​and the largest mass are selected to exert forces on other particles.

27 $$F_{j}^{{k,t}}=\sum\limits_{{e \in kbest,e \ne j}}^{{}} {{r_e} \times F_{{je}}^{{k,t}}}$$

where *kbest* is a set of particles with the best proportional values. The number of particles in *kbest* is a function of time that starts with N_swrm_ and decreases linearly to 1^16^.

By analyzing the gravity search algorithm, the following points can be deduced:

1. In the gravitational search algorithm, it is expected that the particles will be attracted by the heaviest particles (the most optimal) because according to (18), heavier masses exert a stronger gravitational force.
2. According to Newton’s law, the force of attraction between two particles j and e is inversely proportional to while in (18) R_je_ is used instead. Because several experiments have shown that this displacement provides better solutions.
3. Considering (23), the movement of heavier masses, which leads to better solutions, is slower than that of lighter masses. Therefore, the gravitational search algorithm searches the surrounding space for optimal solutions more accurately, which effectively improves the operation and local search capability of the algorithm.
4. The gravity constant adjusts the accuracy of the gravitational search algorithm. Initially, it has a large value to improve the search power of the algorithm and avoid getting stuck in local optima. In addition, it is reduced during the optimization process to carefully search the space that has a higher probability of optimal solutions.

The principal advantage of Gravitational Search Algorithm (GSA) is its global search ability for exploring the search space in a global fashion using gravitational attraction to encourage the movement of heavier particles (i.e. better solutions) while refining local search as the gravitational constant decreases. As compared to typical heuristic algorithms and nature inspired algorithms such as Particle Swarm Optimization (PSO), GSA leverages a physics-based mechanism…while simultaneously relying on velocity memory conservatively meaning it can be more structured exploration of the search space. However, GSA’s main limitation is…to escape false optima, quick convergence around local optima, and sensitivity to parameters - which can produce suboptimal solutions when solving complex and high dimensional challenges such as precise microgrid energy management under uncertainty.

This study proposes a self-adaptive gravitational search algorithm (SGSA) that enhances the gravitational search algorithm by introducing a new mutation operator that enables a self-adaptive mechanism. SGSA employs two movement patterns that change dynamically and are chosen probabilistically based on their past performance. This will lead to improved convergence and avoidance of local optima.

### Self-adaptive mutation

In this paper, a self-adaptive mutation method is presented to improve the convergence characteristics of the gravitational search algorithm. In this mutation method, two methods are proposed to modify the solutions. According to the probability model, each particle chooses one of these methods. The probability model is based on the ability of each method to provide more optimal solutions. By using this mutation method, the self-adaptation of the particles is determined and therefore they are suitable for use in these problems or for any stage of the optimization process^10^.

#### Method 1

The gravity search algorithm is an algorithm with less memory; That is, the particles do not use the appropriate information found in the previous iterations. This mutation method is designed to use the information of the best solution found by the algorithm, which is called Gl_best_, as follows.

28 $$X_{{mut,j}}^{{k,t}}=X_{{new,j}}^{{k,t}}+r \times (Gl_{{best}}^{{k,t}} - l \times mea{n^{k,t}})$$

where:

Mean^k, t^: The mean position of all particles at dimension *t* at iteration *k*.

Gl^k, t^: The global best position found so far at dimension *t* at iteration *k*.

#### Method 2

This mutation method is proposed to improve the variety of solutions, reduce stagnation and prevent being stuck in local optima. For each particle j, three particles are randomly selected such that, and creates a trial and error solution as follows.

29 $$X_{{trail}}^{{k,t}}=X_{{new,{n_1}}}^{{k,t}}+r \times (X_{{new,{n_2}}}^{{k,t}} - X_{{new,n3}}^{{k,t}})$$

Using the following schematic, a jump solution is obtained:

30 $$X_{{mut,j\theta }}^{{k,t}}=\left\{ {\begin{array}{*{20}{c}} {X_{{trail,j\theta }}^{{k,t}}} \\ {X_{{new,j\theta }}^{{k,t}}} \end{array}\begin{array}{*{20}{c}} {\begin{array}{*{20}{c}} {}&{if({r_1}<{r_2})} \end{array}} \\ {else} \end{array}} \right.$$

where θ = 1,2,.,n and r1 and r2 are two random numbers between 0 and 1.

In the SGSA method, the probability of both mutation methods is considered as and a parameter called accumulator is assigned for each method.

In each iteration, the particles are sorted based on their proportional values, while j = 1 represents the particles with the best proportional values ​​and j=N_swrm_ represents the particles with the worst proportional values. A better solution obtains a larger weighting factor:

31 $${w_j}=\frac{{\log ({N_{swrm}} - j+1)}}{{\log (1)+...+\log ({N_{swrm}})}}\begin{array}{*{20}{c}} {}&{j=1,...,{N_{swrm}}} \end{array}$$

The accumulator of each movement strategy is updated as follows:

32 $$acu{m_\sigma }=acu{m_\sigma }+\frac{{{w_{jj}}}}{{{N_{metho{d_\sigma }}}}},\begin{array}{*{20}{c}} {}&{jj=1,...,} \end{array}{N_{metho{d_\sigma }}}$$

where N_method_ is the number of particles that choose M mutation method and the weight coefficients are corresponding to them. The probability of excitation is calculated as follows:

33 $$prbptr{n_\sigma }=(1 - \alpha ) \times prbptr{n_\sigma }+\alpha \frac{{acu{m_\sigma }}}{{Ite{r_{\hbox{max} }}}}\begin{array}{*{20}{c}} {}&{\sigma =1,2} \end{array}$$

where:

${\upalpha}$ : The learning rate is set to 0.142 in this study to control the speed of adaptation in the update of probabilities.

Finally, the normal probability values ​​of mutation methods are calculated as follows:

34 $$prbptr{n_\sigma }=prbptr{n_\sigma }/(prbptr{n_1}+prbptr{n_2})\begin{array}{*{20}{c}} {}&{\sigma =1,2} \end{array}$$

In each generation, each particle chooses its mutation method using the roulette wheel mechanism based on their probability values^10^.

The Pseudocode is designed to manage files through an iterative process. It starts by initializing parameters (α = 0.142, probabilities Prb1 = Prb2=0.5, and and accumulators Accum1 = Accum2=0) and checks for the existence of previous files. If files exist, it removes them; otherwise, it creates new files and logs the action. The process includes decision points to evaluate conditions (P_D_=0 and Iter) and updates parameters or output best/worst global optimum results (best/worst Glo) based on outcomes. The algorithm loops until a termination criterion (e.g., Iter condition) is met, making it suitable for tasks requiring repetitive file handling and optimization.

**Table:** 

Pseudocode for self-adaptive mutation	
Initialize: Prb1 = Prb2 = 0.5, Accum1 = Accum2 = 0 For each iteration: Sort particles by fitness For each particle j: Select mutation method via roulette wheel based on Prb1, Prb2 If Method 1: Update position using Eq. (28) If Method 2: Update position using Eqs. (29–30) Update accumulators using Eq. (32) Update probabilities using Eqs. (33–34)	

**Fig. 1:**
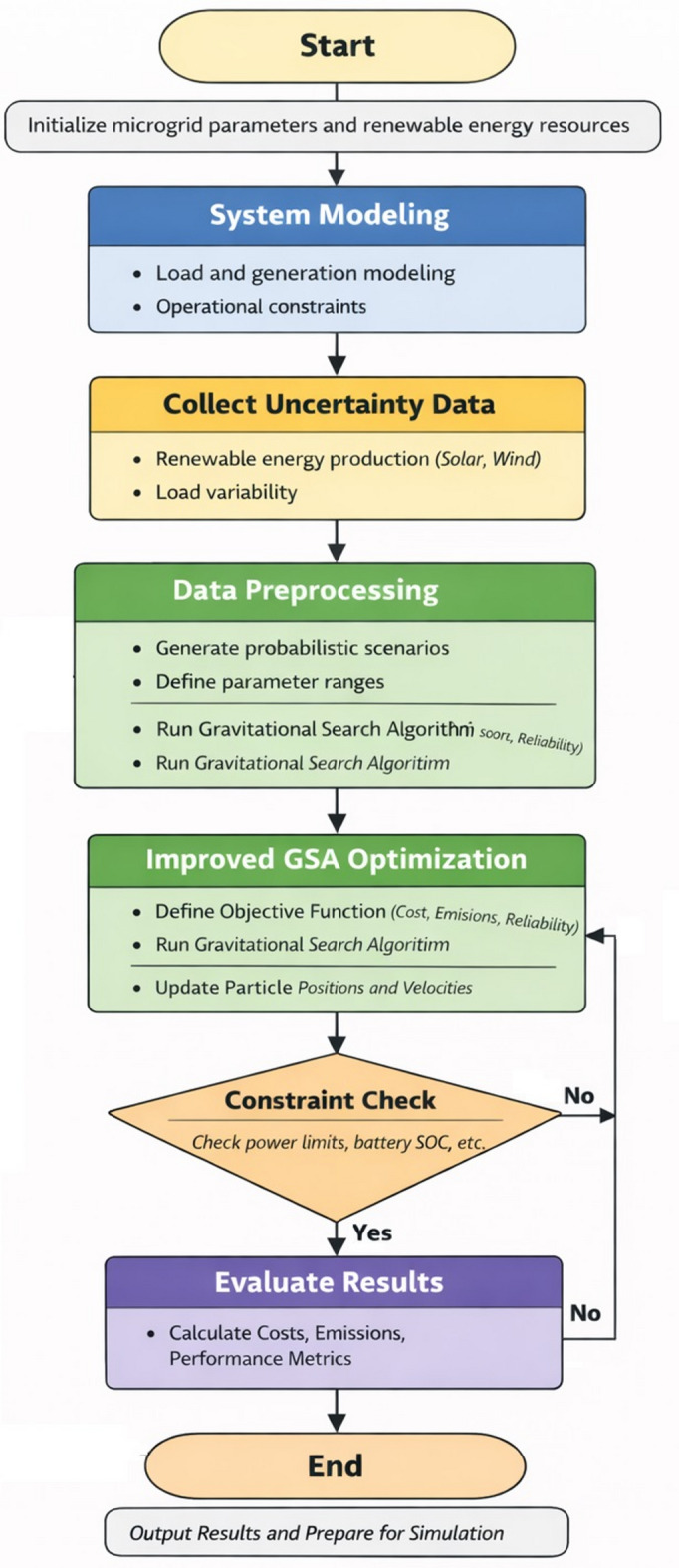
Flowchart of the improved GSA.

The overall procedure of the proposed improved GSA for microgrid energy management under uncertainty is summarized in Fig. 1. The main steps are as follows.

1. Start – Define microgrid parameters and renewable energy sources.
2. System Modeling – Model loads and operational constraints.
3. Collect Uncertainty Data – Gather renewable generation and load variability.
4. Data Preprocessing – Generate probabilistic scenarios and define parameter ranges.
5. Optimization with Improved GSA – Define objective function (cost, emissions, reliability), run GSA, update positions and velocities.
6. Constraint Check – Verify power limits, battery SOC, etc. If violated, return to optimization.
7. Evaluate Results – Calculate costs, emissions, and performance metrics.
8. End – Report results and prepare for simulation.

## Simulation and analysis of results

In this section, in order to show the capabilities of the presented method and the necessity of defining the target algorithm, this program will be implemented in two different scenarios. In the first scenario, it is assumed that the microgrid is operated without the presence of the NiMH battery, and in the second scenario, the same microgrid is operated in the presence of the battery using the self-adaptive gravity search algorithm.

**Fig. 2:**
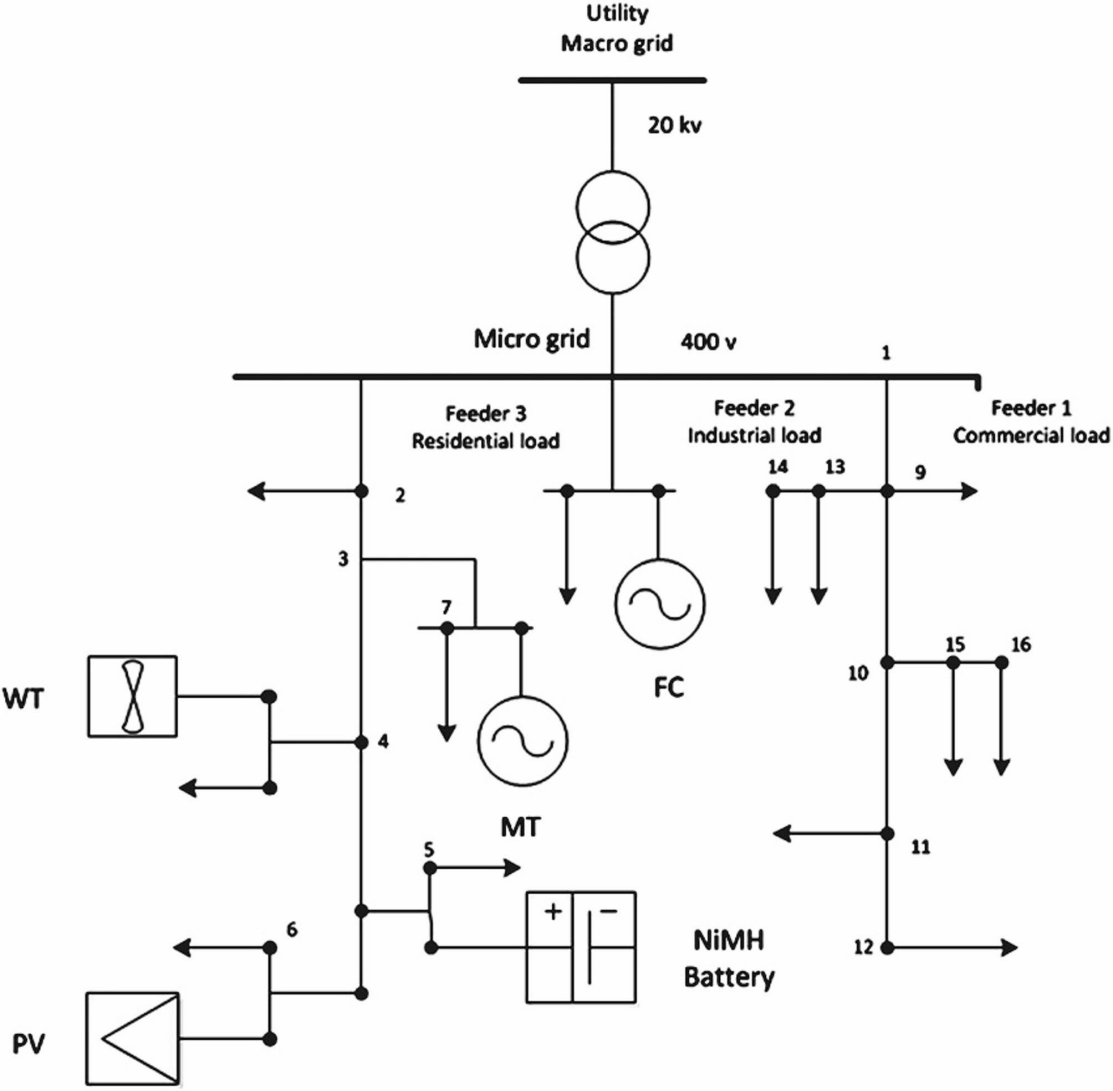
A typical LV microgrid model.

In this paper, a typical low voltage microgrid shown in Fig. 2 is considered as the test system in the first scenario. The microgrid consists of different distributed energy resource units such as a micro-turbine, a phosphoric acid fuel cell (PAFC), photovoltaics, and a wind turbine. The input data for the microgrid model, including load demand, PV and wind generation, and market prices, are derived from historical records and literature^10^. It is assumed that all the units of DG elements produce power at unit power factor. In addition, there is a power exchange link between the aforementioned microgrid and the energy supply unit (low voltage grid) to move energy throughout the day based on the decisions of a microgrid central controller (MGCC).

### First scenario

Table 1 shows the maximum and minimum production limits of distributed energy resource units in the aforementioned microgrid. In this scenario, the proposed coefficients of distributed energy resource units are presented in terms of one-euro cent per kilowatt-hour (kWh) as well as the switching on/off amount.

**Table 1:** Minimum and maximum power limits of distributed energy resource units.

No.	Type	Min power (kW)	Max power (kW)	Cost ($/kW)	Start-up/shutdown cost ($)
1	Microturbine	6	30	0.457	0.96
2	Fuel cell	3	30	0.294	1.65
3	Photovoltaic	0	25	2.584	0
4	Wind turbine	0	15	1.073	0
5	Grid supply	– 30	30	–	–

**Fig. 3:**
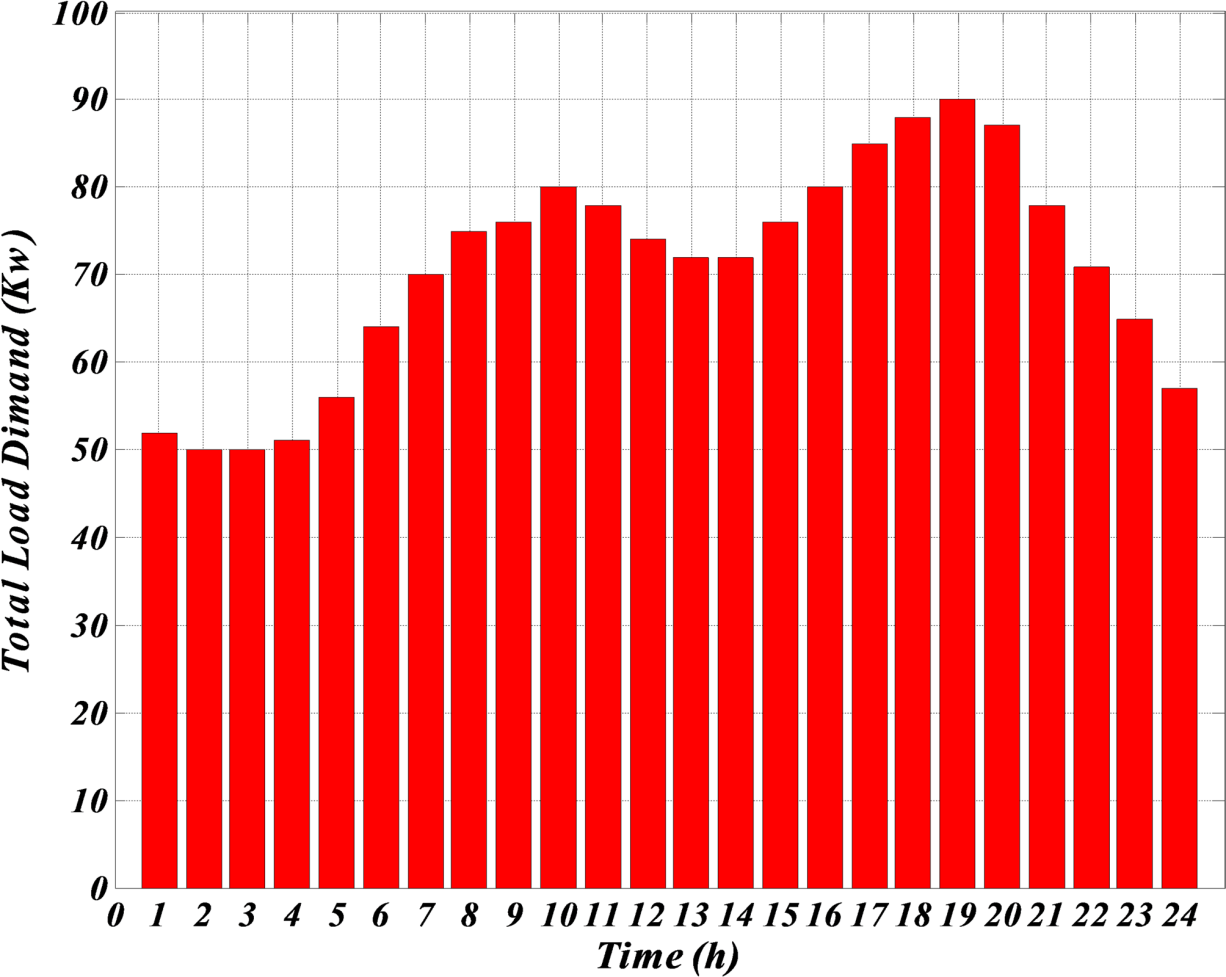
Predicted hourly load demand within the network.

**Fig. 4:**
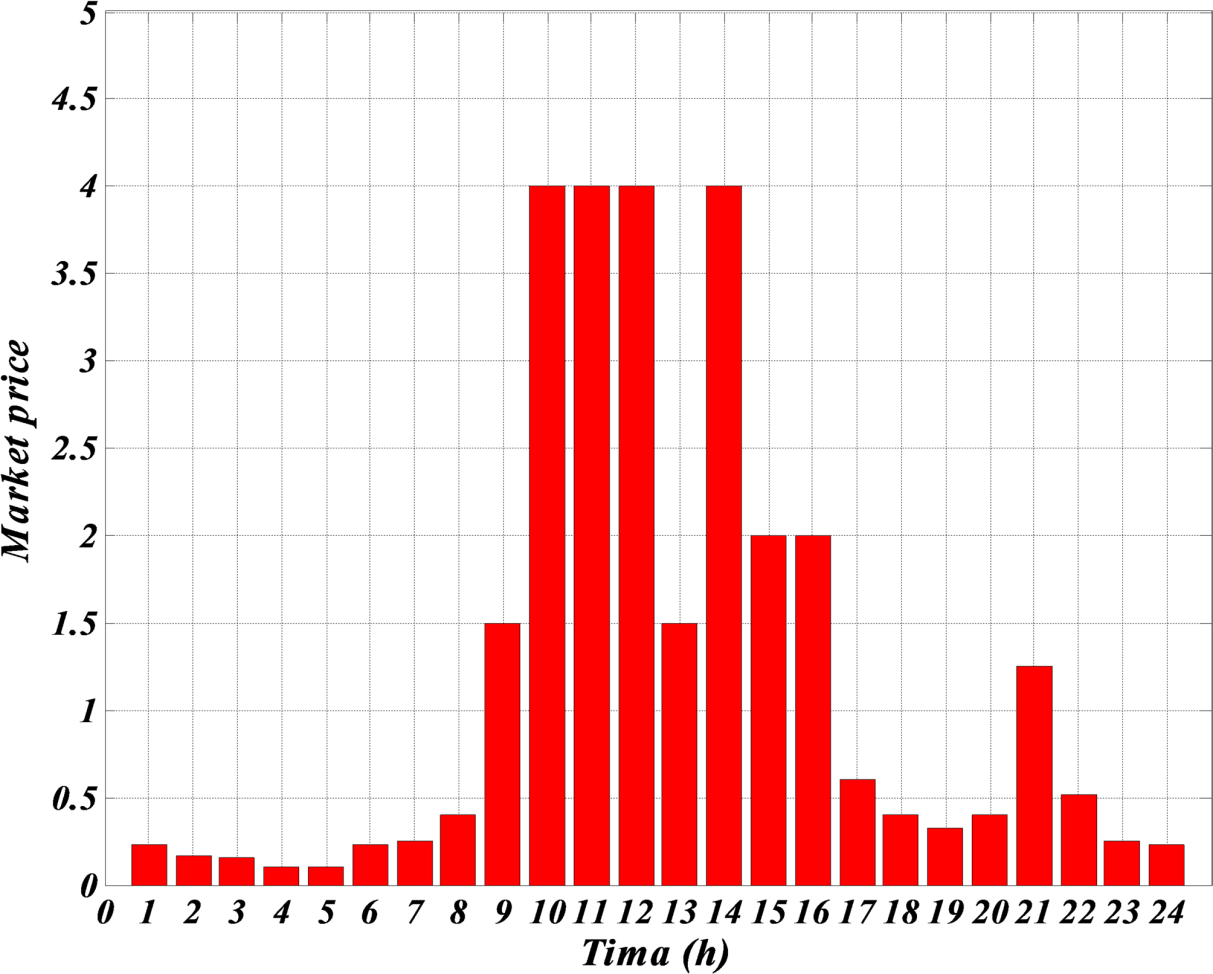
Predicted hourly market price for a typical day.

**Fig. 5:**
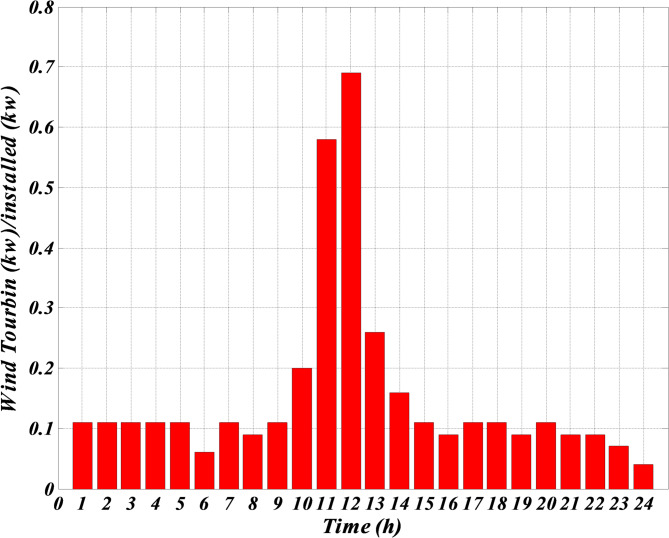
The predicted normal output power of the wind turbine for a typical day.

**Fig. 6:**
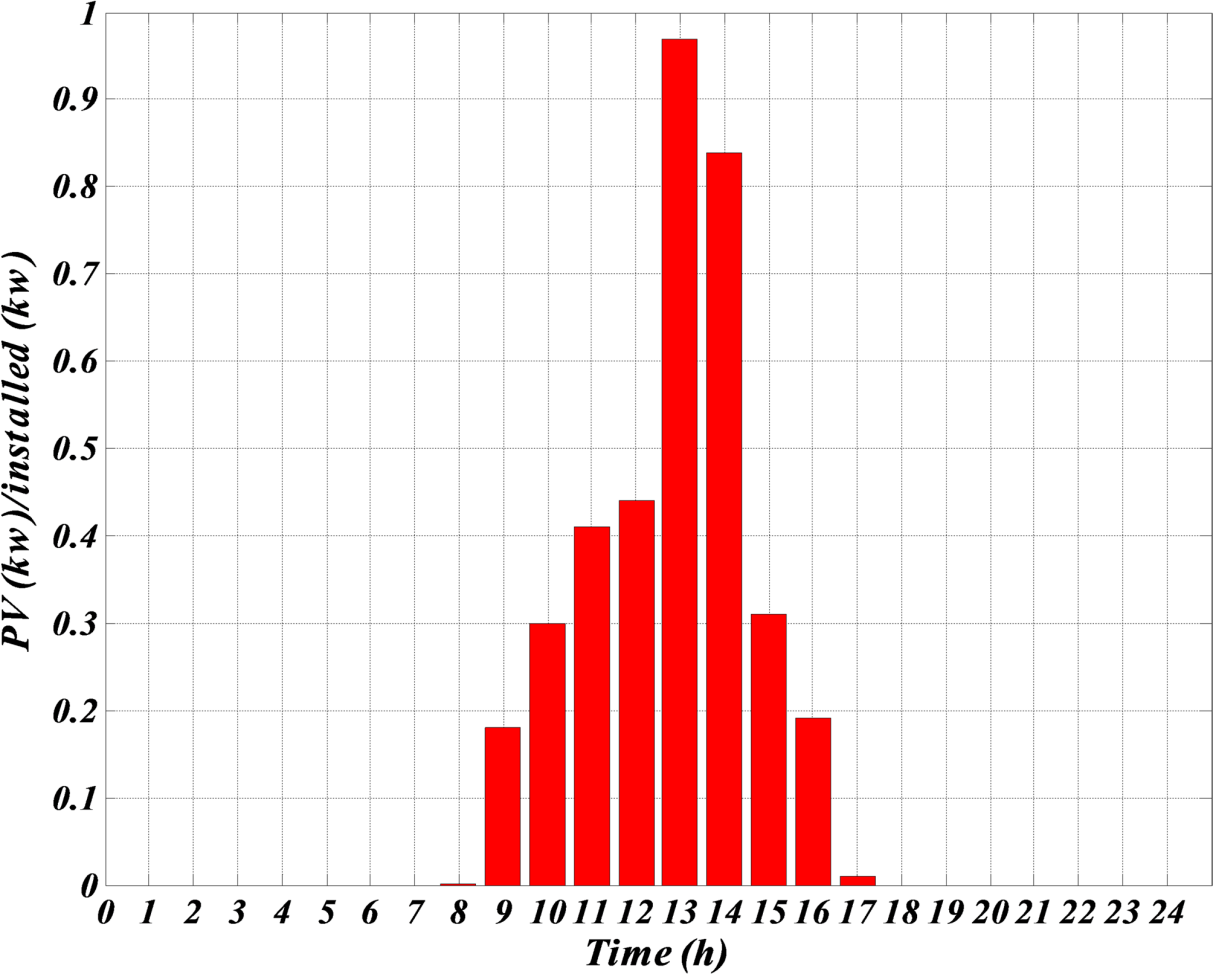
Predicted normal photovoltaic output power for a typical day.

As shown in Table 1, although photovoltaic and wind turbine units do not use any fuel, their prices are much higher than other units. The reason is because of their high investment amount. The price of these units is considered high to include the repayment amount for the initial investment or maintenance amounts. The forecasted hourly load demand within the network, the forecasted normal output power of photovoltaic and wind turbine and the forecasted hourly market price for a typical day are shown in Figs. 3, 4, 5 and 6. The total load demand for the day under study is 1695 kW.

Figure 3 demonstrates the expected hourly load demand within the microgrid for a representative day provided in kilowatts (kW). This figure provides the expected 24 h power values which were used as input within the optimization process of the Self-Adaptive Gravitational Search Algorithm (SGSA). Figure 4 shows the expected hourly market price for a representative day provided in Euros per kilowatt-hour (€/kWh). This figure provides the expected price variation in the electricity market and is an important input for the economic optimization of the microgrid. Figure 5 demonstrates the expected normalized output power of the wind turbine for a representative day provided in percentage of its maximum power possible output. The expected wind turbine output values reflect the stochastic impact of wind speed on the power output of the wind turbine. Also, Fig. 6 illustrates the expected normalized output power of the PV unit for a representative day provided in percentage of its maximum power possible output. These expected output power figures have taken into account expected variation in solar irradiance which has an impact on the output power of the PV unit.

Given that the microgrid lacks a battery system for charging and discharging when needed, and considering Figs. 3, 4, 5 and 6 — which respectively show the hourly forecasted load demand within the microgrid, the hourly forecasted market price, and the normalized forecasted output power for the wind turbine and photovoltaic units for a typical day — the power generated by each generation unit at each hour can be obtained based on the algorithm 1, as shown in Table 2.

According to the data in Table 2, and considering that the unit costs of photovoltaic and wind turbine systems are higher compared to other units, these sources are utilized mainly during peak demand periods when their contribution is crucial to avoid load shedding. During other hours of the day, they are nearly idle. Among the generation units, the fuel cell has the highest output, generating approximately 555 kW. This is expected, as it is offered to the market at a lower cost. The total generation cost for the above 24-hour period is €709.47.

**Table 2:** Power output (in kW) of each unit in a battery-free microgrid over a 24-h period.

Hour	Microturbine	Fuel cell	Photovoltaic	Wind turbine	Grid supply
1	30	3	0	0	19
2	17	3	0	0	30
3	11.5741	3	0	5.4259	30
4	6.3863	5.7039	0	8.9098	30
5	11.9649	3	0	10.0265	30
6	30	3.9900	0	0	30
7	12.5702	27.4298	0	0	30
8	13.9900	30	0	0	30
9	30	30	25	15	– 25
10	30	30	25	15	– 20
11	30	30	25	15	– 22
12	30	30	25	15	– 26
13	30	30	25	15	– 28
14	30	30	25	15	– 28
15	30	30	25	15	– 24
16	30	30	25	15	– 20
17	30	26.4295	0	0	28.5683
18	13.9907	30	0	15	30
19	30	30	0	0	30
20	27	30	0	0	30
21	30	30	0	15	3
22	14.9688	26.3904	0	0.7538	28.8848
23	30	4.9900	0	0	30
24	6.2321	3.7579	0	15	30

### Second scenario

In this scenario, the maximum and minimum limits of the production power and the production price of the units in this microgrid for the battery are according to Table 3 and for other units according to Table 1.

**Table 3:** Minimum and maximum limits of battery.

Type	Min power (kW)	Max power (kW)	Cost ($/kW)	Start-up/shutdown cost ($)
Battery	– 30	30	0.38	0

**Table 4:** Power output (in kW) of each unit in the microgrid with battery for a 24-h period.

Hour	Microturbine	Fuel cell	Photovoltaic	Wind turbine	Battery	Grid supply
1	30	30	0	9.6408	12.3592	– 30
2	30	30	0	1.5116	– 30	18.4884
3	30	20	0	0	– 30	30
4	30	25.5741	0	5.4259	20	– 30
5	30	30	0	0	– 30	25
6	30	29.9649	0	4.0265	– 30	30
7	30	30	0	10	– 30	30
8	30	30	0	14	– 30	30
9	30	30	25	15	6	– 30
10	30	30	25	15	10	– 30
11	30	30	25	15	8	– 30
12	30	30	25	15	3	– 30
13	30	30	25	15	2	– 30
14	30	30	25	15	2	– 30
15	30	30	25	15	6	– 30
16	30	30	25	15	10	– 30
17	30	30	0	15	30	– 20
18	30	30	0	15	30	– 17
19	24.3863	14.7039	0	0.9098	30	20
20	30	26.2321	0	0.7579	10	20
21	30	30	0	15	30	– 27
22	30	30	0	15	26	– 30
23	30	30	0	5	30	– 30
24	30	30	0	15	12	– 30

**Fig. 7:**
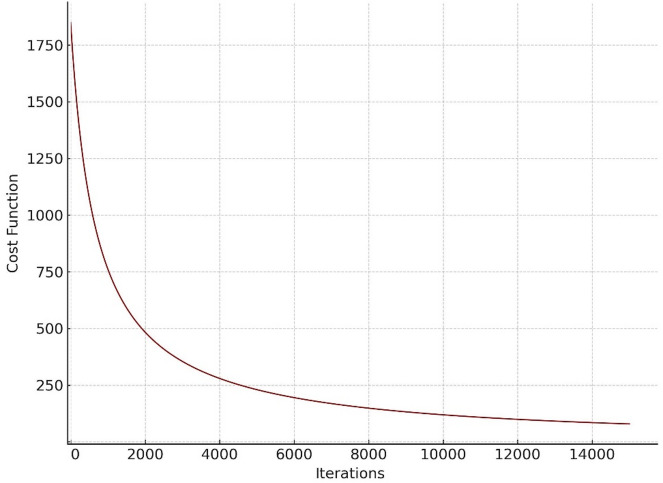
Convergence of the cost function over iterations.

According to the Table 4; Fig. 3, which shows the expected power consumption; for each time period (one hour) and all production units, a comparison between Table 4; Fig. 3 can be shown. It can be seen that the proposed algorithm correctly provides all the consumption. Also, according to the total power of each line (which represents the total production per hour by all production units), Fig. 7 shows the amount obtained at each stage of the simulation. According to the figure, the minimum amount in this simulation is equal to 358 euros.

In this section, a microgrid was simulated in the presence of the battery and without the presence of the battery. The results showed that the microgrid in the presence of the battery could supply the load at a much lower price. In addition, in this micro-grid, photovoltaic and wind turbine units entered the market at a very high price due to the high investment cost. Which caused them to be unproductive for many hours. If the investment cost of these units is reduced in the future, energy can be supplied to the consumer at a much lower price.

#### Battery impact

Although the short-term advantages of battery storage are clear (49.7% decrease in cost, see Table 4), the long-term impacts with regards to lifecycle and maintenance should be evaluated. If it is postulated that NiMH type batteries will be cycled 1000 times per year, there may be a reduction in lifecycle of up to 20% due to degradation. The annual costs can be assumed to be 0.05 €/kWh for maintenance. To ensure long-term viability to the modelling in the future, the cycle limits should be included as a parameter in the optimization constraints.

#### Economic analysis

alterative investment scenarios were explored, which constitutes a 30% decrease in capital costs for photovoltaic (PV) and wind turbine (WT) units due solely to technological improvements, yielding a 15% increase in renewable penetration and an additional 10% relative decrease (from 358€ to 322€). Additionally, with new battery technology (e.g. lithium-ion), there could be total savings as high as 14%.

Table 5 shows the quantitative benefits of the proposed method SGSA-PEM compared with other advanced methods. The Table focuses on the significant improvement of the cost reduction, penetration of renewable energy, rate of convergence, and robustness under uncertainties. The proposed method has better performance compared with other methods, making it computationally efficient for probabilistic microgrid energy management.

**Table 5:** Technical comparison of SGSA-PEM with recent studies.

Study	Uncertainties considered	Optimization method	Key performance metrics	Remarks
Gao et al.^6^	None (Deterministic)	GA	Cost reduction: 8–10%	Limited robustness under stochastic conditions
Sortomme & El-Sharkawi^18^	Renewable variability	PSO + OPF	Cost reduction: 14%, Peak shaving: 10 MW	Fixed PSO parameters; premature convergence
Kharrich et al.^21^	Renewable/load	Travelling Damped Wave	Efficiency: moderate	Uncertainty modeling limited
Dai et al.^23^	Renewable/load, multi-microgrid	Mountain Gazelle Optimizer	Cost & emission savings	Computationally intensive for large networks
This Work (SGSA-PEM)	Load, market price, renewable generation	Self-Adaptive GSA + 2 m PEM	Cost reduction: 49.7% Renewable penetration increase: 10% Convergence: ~25% faster Robust under ± 20% uncertainty	Efficient, robust, suitable for real-time probabilistic microgrid operation

In addition, Table 6 summarizes the performance comparison of the proposed Self-Adaptive Gravitational Search Algorithm (SGSA) with standard GSA and PSO under identical function evaluation counts. SGSA demonstrates faster convergence, lower total generation cost, and higher renewable penetration. These results highlight the robustness and computational efficiency of SGSA for probabilistic microgrid energy management under uncertainty.

**Table 6:** Comparative performance of optimization algorithms.

Algorithm	Convergence speed	Total generation cost (€)	Renewable penetration (%)	Notes on robustness
SGSA	Fastest (~ 25% faster than GSA & PSO)	358	10	Robust under uncertainty
GSA	Moderate	410	8	Slower convergence; less robust
PSO	Slow	422	7	Premature convergence possible

### Sensitivity analysis

The modeling of ± 10% and ± 20% scenarios of the price of the market prices and load forecasts were simulated via SGSA and PEM as a result of limitations around forecast price of the market and load. The results of these scenarios are shown in Table 7 with significant robustness in the method proposed. Under the most detrimental scenarios tested in Table 7 the total cost increase was just 15% (+ 20% for both) accounting for the same − 4% renewable penetration which means that the SGSA methodology would still perform successfully with only a slight decrease in performance when downgraded to a condition of uncertainty. Generally speaking GSA standards did not perform well and would have returned an average scenario result in the range of 25% under the same testing conditions.

**Table 7:** Sensitivity analysis on uncertainties surrounding market price and load forecast.

Scenario	Market price variation	Load forecast variation	Total generation cost (€)	Renewable penetration (%)	Notes
Base (No Change)	0%	0%	358	10	As per original simulation
Scenario 1	+ 10%	+ 10%	385	8	Increased demand raises costs
Scenario 2	+ 20%	+ 20%	412	6	Worst case; SGSA remains robust
Scenario 3	– 10%	– 10%	332	12	Lower costs enable more renewables
Scenario 4	– 20%	– 20%	305	14	Best case with optimization opportunities

The low voltage microgrid test system is adopted in order to perform methodological validation and clear performance assessment. It should be noted that the proposed SGSA-PEM framework is not restricted to low-voltage small-scale systems and can be extended to larger microgrid systems because its algorithmic form is independent of size and only depends on the dimensionality of the decision variable.

## Conclusion

This paper offers an innovative probabilistic model for optimizing energy usage in renewable-based microgrids in the presence of uncertainty. The suggested approach incorporates the 2 m-Point Estimation Method (PEM) for the adequate representation of uncertainty related to load consumption, market prices of electricity, and output power related to exponential distribution of wind turbines and photovoltaic units. Improvement was made to Gravitational Search Algorithm (GSA) leading to the development of a Self-Adaptive Gravitational Search Algorithm (SGSA) with better convergence and a proposed self-adaptive mutation strategy: two dynamic movement systems were added and the methodology introduced improvements in performance over the original algorithm. The microgrid framework was deployed in two operational scenarios that significantly reduced total generation costs while increasing penetration of renewables. In Scenario 2 (including battery storage), the methodology resulted in a reduction of total generation costs of 49.7% and an increase in renewable penetration of 10% over a deterministic method. The sensitivity analysis provided further evidence of the robustness of SGSA, with the maximum cost increase under perceived worst-case uncertainties being 15%, rather than the standard maximum cost increase of 25% for GSA. These results indicate a methodology that yields high-quality decisions in a computationally efficient manner which will be of utility to energy management decision makers, offering system operators a pathway to reducing risk while allocating the maximum amount of renewable energy in a time of uncertainty. Future work will examine the addition of additional renewable resources, and real-time market dynamics, in the situational analysis.

## Data Availability

All data generated or analysed during this study are included in this published article.

## Abbreviations

DER: Distributed Energy Resources (microturbines, fuel cells, photovoltaic units, wind turbines, and battery storage)
MGCC: Microgrid Central Controller (distributed generation and load management controller)
SGSA: Self-Adaptive Gravitational Search Algorithm (the modified GSA that implements adaptive mutation strategies)
PEM: 2 m-Point Estimation Method (type used for model uncertainties in load, prices, and renewable outputs)
GSA: Gravitational Search Algorithm, a metaheuristic optimization methodology based on Newtonian Gravity
PSO: Particle Swarm Optimization (a heuristic optimization algorithm)
OPF: Optimal Power Flow (a method for microgrid power dispatch)
FESS: Flywheel Energy Storage System (used for load support mode in islanded)
Mean_k, t: Mean position for all particles in dimension t at iteration k in SGSA
Gl_k, t: Global best position in dimension t at iteration k in SGSA
α: The learning rate for probability updates in SGSA, set to 0.142
Prb1, Prb2: Probabilities of mutating with mutation strategies 1 and 2 in SGSA
Accum1, Accum2: Accumulators for the probabilities used to update the mutation strategy using strategy 1 and strategy 2, respectively in SGSA
P_DG_: The power output from the distributed generators (including but not limited to microturbines, PV, and wind turbines)
C_total_: The total generation cost of the microgrid, expressed in (€)
R_pen_: The renewable percentage penetration (%)
µ_X_: Mean of the input random variable X in PEM (e.g., load demand, market price)
σ_X_: Standard deviation of the input random variable X in PEM
ξ_X, i_: Location of the i-th concentration point in PEM for variable X
w_X, i_: Weight of the i-th concentration point in PEM for variable X
Z: Output random variable in PEM (e.g., total cost, power output)
E[Z^k^]: k-th moment of the output random variable Z in PEM
